# Temperature-Modulated Scanning Calorimetry of Melting–Recrystallization of Poly(butylene terephthalate)

**DOI:** 10.3390/polym13010152

**Published:** 2021-01-01

**Authors:** Akihiko Toda

**Affiliations:** Graduate School of Advanced Science and Engineering, Hiroshima University, Higashi-Hiroshima 739-8521, Japan; atoda@hiroshima-u.ac.jp

**Keywords:** melting, crystallization, recrystallization, poly(butylene terephthalate), periodic temperature nodulation

## Abstract

The melting and recrystallization behaviors of poly(butylene terephthalate) (PBT) were investigated using temperature-modulated scanning calorimetry in both fast- and conventional slow-scan modes. With this method, the response of multiple transition kinetics, such as melting and recrystallization, can be differentiated by utilizing the difference in the time constants of the kinetics. In addition to the previous result of temperature-modulated fast-scan calorimetry of polyethylene terephthalate (PET), the supporting evidence of another aromatic polyester, PBT, confirmed the behavior of the exothermic process of recrystallization, which proceeds simultaneously with melting on heating scan in the temperature range of double melting peaks starting just above the crystallization temperature up to the main melting peak. Because the crystallization of PBT is much more pronounced than that of PET, similar behavior of recrystallization was obtained by the conventional temperature-modulated differential scanning calorimetry at a slow-scan rate.

## 1. Introduction

**Figure 1:**
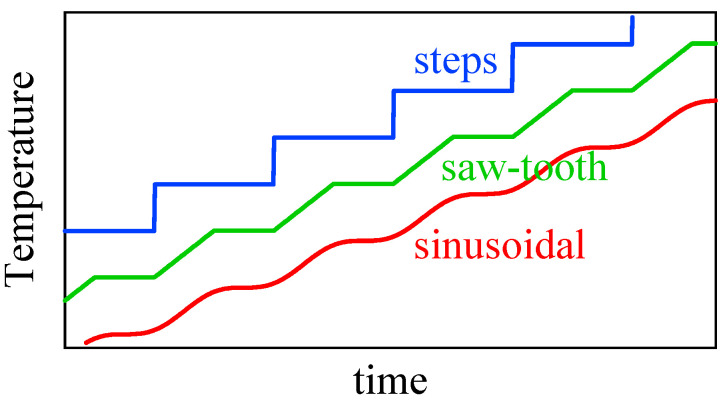
Various types of periodic modulation in temperature of a temperature-modulated scanning calorimetry for the case of heating at constant rate under the condition of heating only.

**Figure 2:**
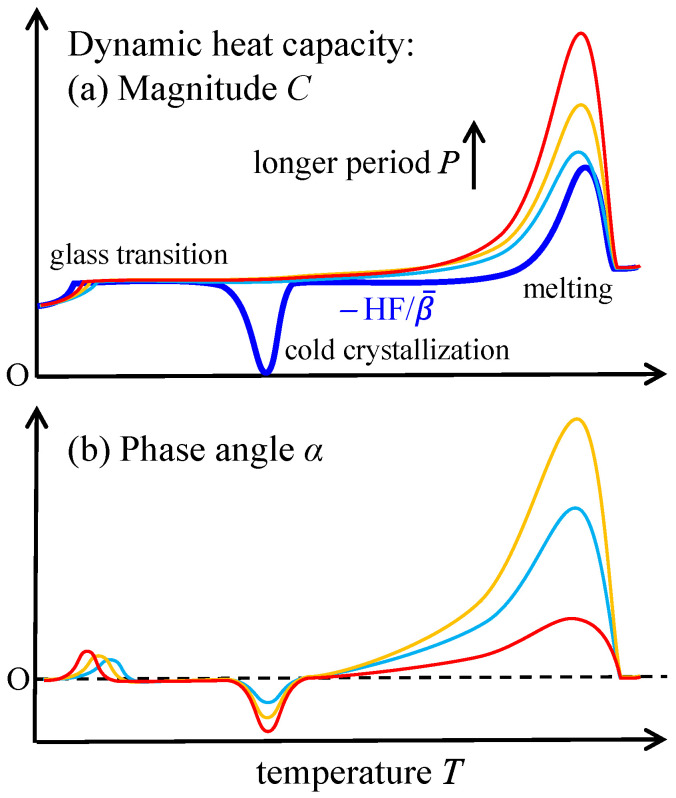
Schematic drawing of typical responses of glass transition, cold crystallization and melting to a periodic temperature modulation, which appear in (**a**) *C* and (**b**) α and show the indicated dependences on applied modulation period. In (**a**), thick line represents $-\mathrm{HF}/\bar{\beta }$.

Temperature-modulated scanning calorimetry is a method that applies a periodic modulation in temperature to the heating or cooling temperature scan at a constant rate or isothermal holding [1]; typical modes of modulation are shown in Figure 1. The response appearing in the heat flow is comprised of the change in the amplitude and the time lag. A dynamic heat capacity of complex quantity $Ce^{-i\alpha }$ can be determined from the response with the magnitude C and the phase angle α determined from the amplitude and the time lag. The method has been mainly employed in glass transition to examine the relaxation phenomena as temperature and frequency dispersions [2]. Because temperature modulation also influences the degree of supercooling or superheating of the kinetics of first-order phase transitions, we can obtain valuable information regarding the transition kinetics [3,4,5,6,7,8,9,10] such as crystallization [3,9], melting [4,6,9], and solid-solid phase transitions [7,10] of polymers [3,4,6,7,8], water [9], and metal alloy [10]; the heating only condition of Figure 1 is utilized to examine the one-way irreversible transition on heating. The typical responses are schematically shown in Figure 2 for the case of crystalline polymer, which is prepared by quenching and in the state of glass before the heating run.

In Figure 2, an effective dynamic heat capacity is shown with the contribution of the processes of glass transition, cold crystallization, and melting, which are identified in Figure 2a by the respective changes in $-\mathrm{HF}/\bar{\beta }$ with the mean heat flow HF divided by the mean underlying heating rate $\bar{\beta }$ and converted to an apparent heat capacity.

(1) glass transition: a step wise change in both of $-\mathrm{HF}/\bar{\beta }$ and C and a peak in α. The dependences on applied period of modulation characterize the relaxation phenomena of the glass transition.
(2) cold crystallization on heating from the glass: large exothermic peak in $-\mathrm{HF}/\bar{\beta }$, very small change in C, and a negative peak in α.
(3) melting region: large peaks in $-\mathrm{HF}/\bar{\beta }$, C, and α.

Those responses of the kinetics of first-order phase transitions of crystallization and melting are characterized by the time constants τ of the kinetics, which determine their dependences on applied modulation period P, approximated in the following manner [4,5,6],

(1) $$Ce^{-i\alpha }=C\cos\alpha -iC\sin\alpha =C_{S}+\left(-F/\bar{\beta }\right){\left[1+i\omega \tau \left(\bar{\beta }\right)\right]}^{-1}$$

where $C_{S}$ represents the sample heat capacity, F the heat flow rate of transition with negative sign for endothermic process, i imaginary unit, ω=2π/P the angular frequency of modulation, and $\tau \left(\bar{\beta }\right)$ the time constant depending on the underlying heating rate $\bar{\beta }$. 

Firstly, very small change in C on cold crystallization suggests that the time constant of crystallization kinetics is much longer than the applied modulation period; it is reasonable because the completion of crystallization usually needs much longer time than the applied modulation period. Then, the dependence of α on the period P becomes monotonically increasing as expected from Equation (1). On the other hand, the negative peak in α suggests that the cold crystallization is a process under deep non-equilibrium state and this exothermic process becomes faster on heating [4]. 

Secondly, the strong dependence on the applied modulation period of the broad and large peaks of C and α on melting suggests that the time constant of the melting kinetics is in the range of the applied modulation periods. Actually, the dependence of α on applied modulation period becomes non-monotonic. On the other hand, as a limiting behavior for P→∞ (ωτ≪1), C approaches to the sum of true heat capacity $C_{S}$ and the heat flow rate of transition divided by $\bar{\beta }$, namely $-F/\bar{\beta }$, as shown in Equation (1) [4]. Therefore, C is monotonically increasing with longer P. However, as the consequence of a complex behavior of metastable chain-folded polymer crystals, C in the melting region can become larger than $-\mathrm{HF}/\bar{\beta }$, as shown in Figure 2 [4]. This behavior is caused by an exothermic process of recrystallization that proceeds simultaneously with melting, namely,

(2) HF=(endothermic HF of melting)−(exothermic HF of recrystallization)

With very small response of recrystallization appearing in C as the nature of crystallization, the melting kinetics solely determines the magnitude of C in the melting region, and hence C can become larger than $-\mathrm{HF}/\bar{\beta }$. It is noted that the difference $\mathrm{HF}-\bar{\beta }C$ is named non-reversing heat flow and utilized for discriminating different types of kinetic response in general [1]; this behavior of melting and recrystallization kinetics is one of the examples. 

The method has been employed using conventional differential scanning calorimetry (DSC). As typically shown in Figure 2, the following characteristics of the melting region of polymer crystals has been clarified experimentally [4].

(1) Melting and recrystallization proceed in parallel.
(2) Those processes start at much lower temperature region than the melting peak of $-\mathrm{HF}/\bar{\beta }$.

The recent development of fast-scan calorimetry (FSC) with a chip sensor [11,12] enables the examination of the method at fast-scan rates [2,13,14,15].

As another characteristic feature of complex melting of polymer crystals, double- or multiple-melting peak appears with a number of crystalline polymers [16]. The origin of the peaks has been controversially discussed [17,18] in terms of the possibilities of distinctive true melting peaks as prepared crystals and the consequence of reorganization and/or melting-recrystallization-remelting. The identification of the true melting point of the original crystals is most important to characterize the thermal property of crystalline polymers [16]. 

For crystalline polyesters, such as poly(ethylene terephthalate) (PET) [18], poly(trimethylene terephthalate) [19], and poly(butylene terephthalate) (PBT) [20], by applying fast-scan calorimetry, it has been shown that the higher-melting peak shifts to lower temperature and the two peaks merge into a single peak at sufficiently high heating rates. This behavior indicated that the higher-temperature melting peak appeared as a result of melting-recrystallization-remelting, which became more pronounced at slower heating rates [16]. Then, the lower-temperature melting peak represents the melting of the original crystals formed during the sample preparation. 

As reviewed in the above, we can differentiate melting and recrystallization kinetics having different time constants by using a temperature modulation method. A recent study of temperature-modulated fast-scan calorimetry (T-M FSC) on the double melting peaks of PET [15] supported the above conclusion on the origin of the double melting peaks. T-M FSC revealed an exothermic process of recrystallization that simultaneously proceeds with melting in the temperature range of the double melting peaks, which starts just above the crystallization temperature. 

In the present study, the melting and recrystallization behaviors of PBT in the range of its double melting peaks were examined using both the conventional DSC and FSC with the application of periodic temperature modulation. By changing the heating rate in the range applicable to FSC, we can systematically control the degree of recrystallization and make the melting and subsequent recrystallization more pronounced. On the other hand, PBT crystallizes much faster than PET in orders of magnitude, and the exothermic heat flow can be more pronounced even at slow-scan rates. Therefore, the recrystallization process in the melting region is expected to be observable even under the conventional slow-scan mode.

## 2. Experimental

PBT was provided by the Chemicals Research Laboratories, Toray Industry, Inc. (Tokyo, Japan) The average molecular weight is $M_{w}=58,600\;{g\;\mathrm{mol}}^{-1}$ with $M_{w}/M_{n}=2.25$. The same PBT was examined in prior papers [20,21] in terms of the crystallization and melting kinetics by FSC. The FSC was of Flash DSC 1 (Mettler-Toledo, Greifensee, Switzerland) with a UFS1 chip sensor, and the conventional DSC was of Q100 (TA Instruments, New Castle, DE, USA), both with a refrigerated cooling system. Dry nitrogen gas was purged through the cells at flow rates of 30 and 50 mL min^−1^. Temperature calibration was performed with indium. For the FSC, 5-μm-thick sections were cut from a pellet using an ultramicrotome (Leica Microsystems, Wetzlar, Germany). Moreover, for isothermal crystallization in the FSC, quenching was done at the rate of $2000{\;K\;s}^{-1}$, which was fast enough to prevent crystallization on cooling in the present condition. With the conventional DSC, crystals were formed from the glass, which was prepared by quenching by placing a sample pan of molten PBT directly on an ice crystal.

**Figure 3:**
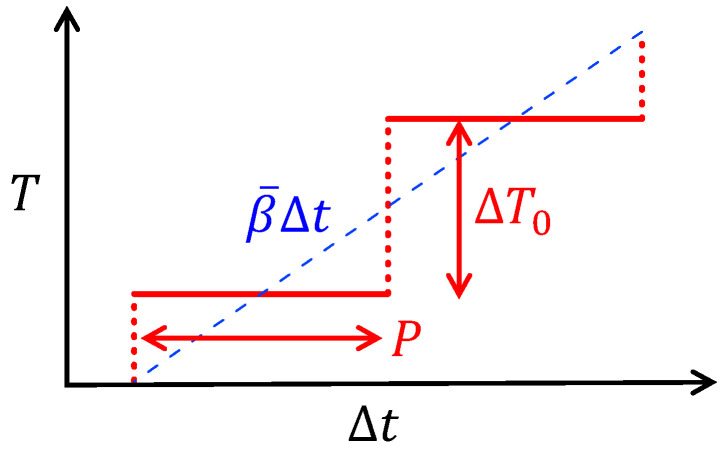
Temperature protocol of periodic temperature jumps with subsequent isothermal holding steps utilized in T-M FSC: $\bar{\beta }=∆T_{0}/P$.

The heating-only mode of periodic temperature modulation was employed with a periodic and stepwise change in the temperature of Flash DSC 1 (Figure 3) and a sinusoidal modulation of Q100. The magnitude C and phase angle α of the dynamic heat capacity of the complex quantity of T-M FSC were determined using the Fourier coefficient of the first harmonic of the heat-flow dataset during each isothermal holding, as described in a prior paper [15]. Those of the T-M DSC were determined using the Q100 instrument. The obtained magnitude C was adjusted by the heat flow at constant heating and cooling outside the melting region. Moreover, a baseline outside of the melting region was subtracted to determine the phase angle α as a response to the transition kinetics. For T-M FSC, PBT samples were examined repeatedly to get the frequency dependence, so that the obtained results are of re-equilibrated PBT in terms of transesterification, which can influence its mean molar mass. 

## 3. Results and Discussion

**Figure 4:**
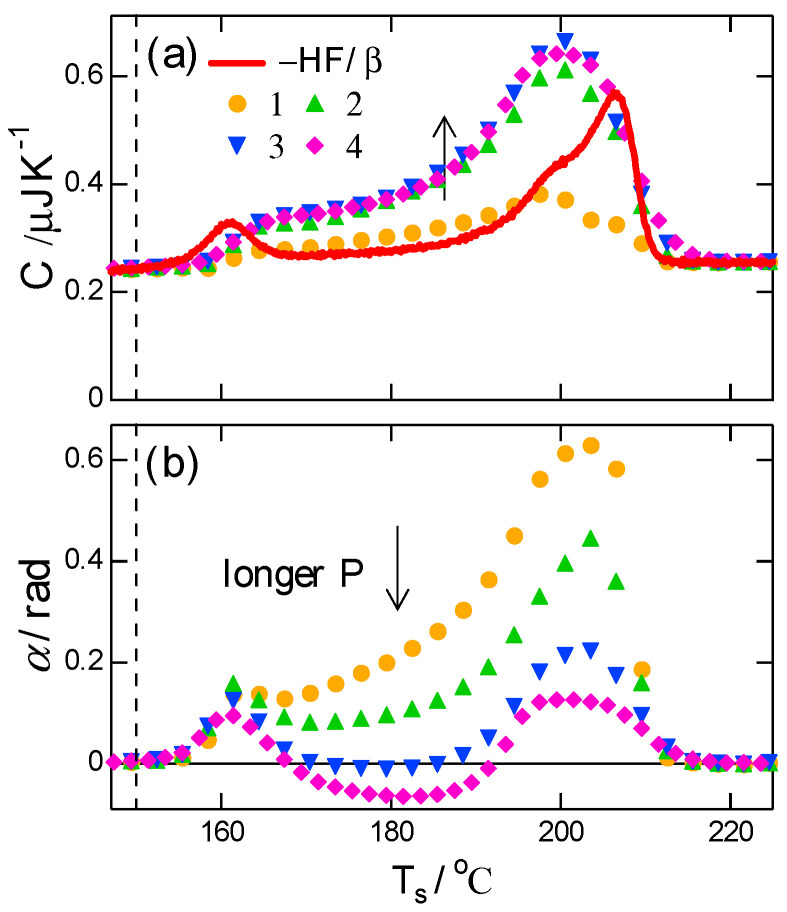
T-M FSC results of (**a**) magnitude and (**b**) phase angle during heating after crystallization at $T_{c}=150\;$°C (vertical broken line) for 5 s. $\bar{\beta }=∆T_{0}/P=100{\;K\;s}^{-1}$. with $\left(∆T_{0}/P\right)/K\;s^{-1}=1:\;3/0.03,\;2:\;6/0.06,\;3:\;9/0.09,\;4:\;12/0.12$. Solid line in (**a**) represents −HF/β.

**Figure 5:**
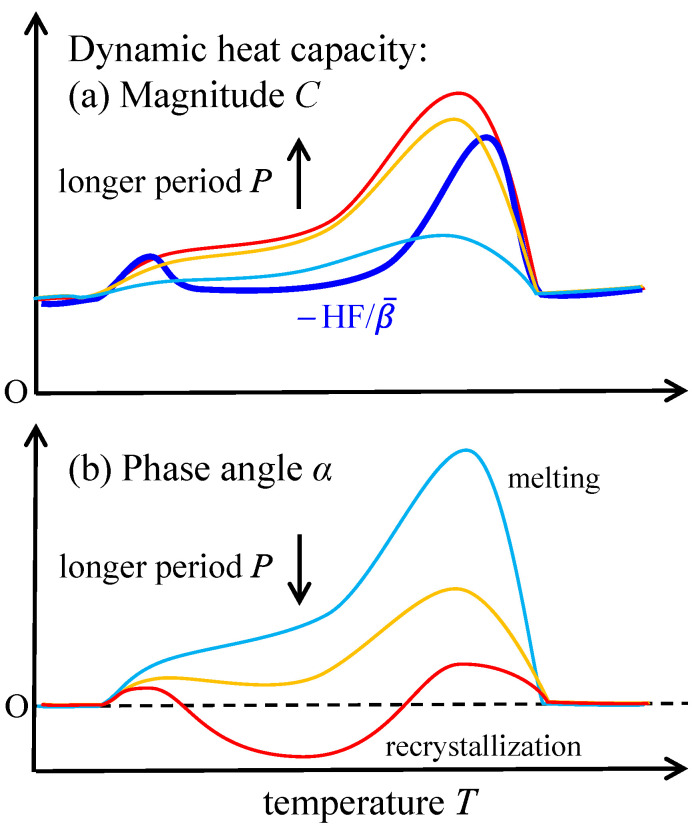
Schematic drawing of the behaviors of (**a**) magnitude and (**b**) phase angle in Figure 4.

The T-M FSC result for the melting region of PBT with double melting peaks is shown in Figure 4, with a mean underlying heating rate $\bar{\beta }=100{\;K\;s}^{-1}$, which was fixed in the T-M FSC shown below, and different modulation periods P in the range of 0.03–0.12 s. Figure 4a,b show the magnitude C and phase angle α of the effective heat capacity, including the contribution of the transition kinetics. The heat flow (HF) was obtained under the same condition without modulation, and it was converted to heat capacity (−HF/β) and included in Figure 4a to show the double melting peaks. In the region of the double melting peaks, the typical characteristics of the T-M scanning calorimetry of polymer crystal melting [15] are confirmed as follows and as shown schematically in Figure 5.

Firstly, the broad peak of C and the corresponding large positive peak of α represent the response of the melting kinetics. Both C and α showed a strong dependence on the applied period P of the temperature modulation, which is characterized by a time constant of the melting kinetics. For longer P, C became larger than −HF/β, suggesting an exothermic process of recrystallization, which proceeds simultaneously with melting. 

Secondly, the broad dip in α with a longer P (Figure 4b) confirms the response of recrystallization, which is characterized by the unusual behavior of a negative peak of α. The appearance of the recrystallization kinetics in α with a longer P is attributed to the difference in the time constants of the kinetics of melting and recrystallization. With a much shorter melting time constant and a longer P, melting follows temperature modulation without delay; therefore, the contribution of the melting kinetics in α approaches zero in an asymptotic manner, as seen in Equation (1). Consequently, with P long enough, the phase angle α is solely determined by the response of recrystallization kinetics which has its time constant longer than P. As shown in the schematic drawing of Figure 2 and Figure 5, the response of recrystallization kinetics brings the negative sign of α because the exothermic kinetics becomes faster at higher temperatures [15]. Therefore, both of the C larger than −HF/β in Figure 4a and the negative peak of α in Figure 4b confirm that the recrystallization process occurred simultaneously with crystal melting of the PBT. 

**Figure 6:**
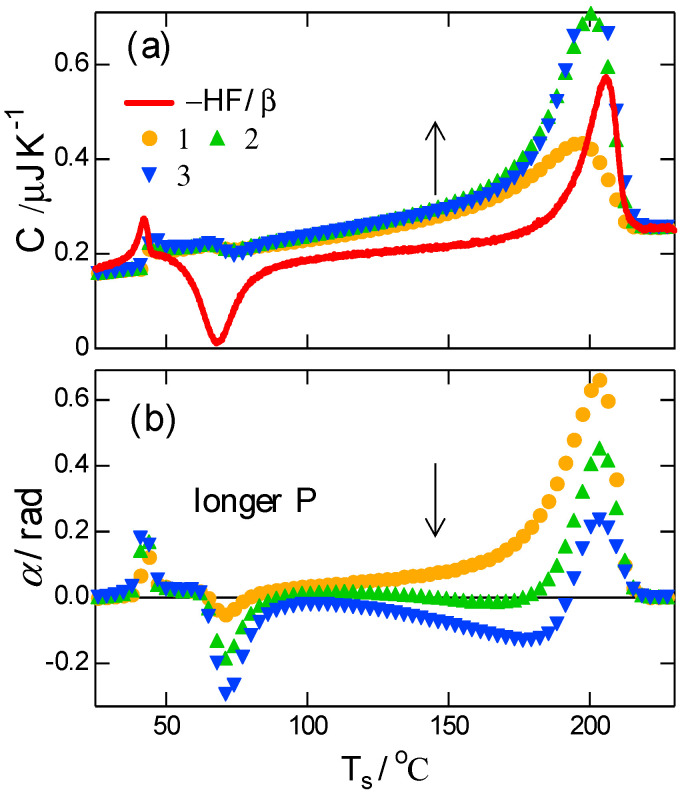
T-M FSC results of (**a**) magnitude and (**b**) phase angle during heating after quenching and annealing at $T_{a}=25\;$°C for $10\;s.\bar{\beta }=∆T_{0}/P=100{\;K\;s}^{-1}$ with $\left(∆T_{0}/P\right)/{K\;s}^{-1}=1:\;3/0.03,\;2:\;6/0.06,\;3:\;9/0.09$. Solid line in (**a**) represents −HF/β.

Figure 6 shows another T-M FSC result of the PBT subjected to quenching. On heating, −HF/β in Figure 6a shows the glass transition, cold crystallization, and melting in that order. Here, the glass transition is characterized by a stepwise increase in C and a positive peak of α with an exothermic peak of enthalpy relaxation in −HF/β. Conversely, cold crystallization is indicated by a small drop in C and a dip in α, which is also a characteristic of recrystallization, as mentioned above. Thereafter, a broad dip in α is discernable with the longest P, and it became deeper at higher temperatures, indicating more pronounced recrystallization at higher temperatures. With the contribution of the melting kinetics, C with longer P became larger than −HF/β, which suggests simultaneous exothermic process of recrystallization over the temperature range much broader than that in Figure 4. The frequency dependence is similar to that in Figure 4.

**Figure 7:**
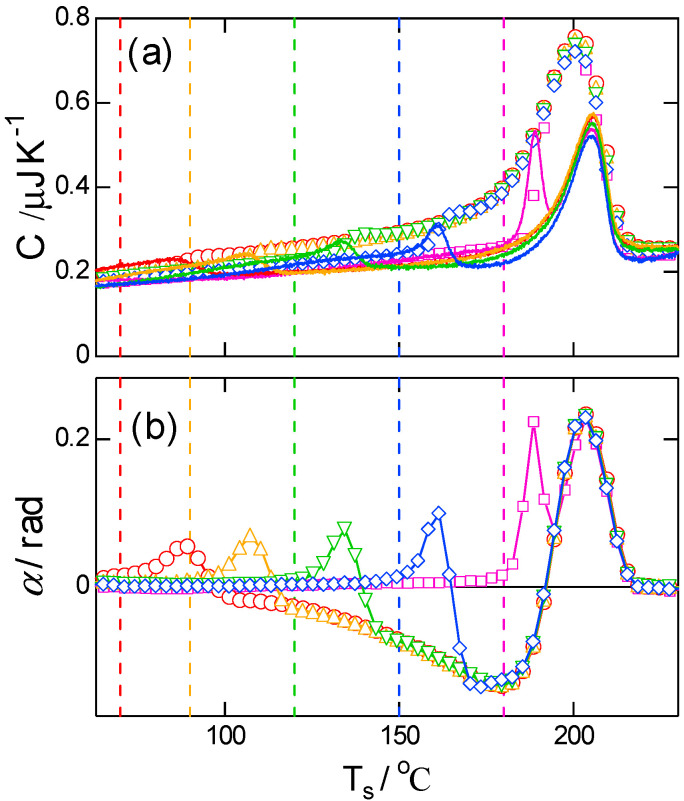
T-M FSC results of (**a**) magnitude and (**b**) phase angle during heating after crystallization at $T_{c}=70\;$°C for $∆t_{c}=5$ s and annealing of $T_{a}=\mathrm{NA},\;90,\;120,\;150,\;180$ °C for $∆t_{a}=5$ s. $\bar{\beta }=∆T_{0}/P=100{\;K\;s}^{-1}$ with $∆T_{0}=9$ K, P=0.09 s. The solid lines in (**a**) represent −HF/β, and the vertical broken lines represent respective treating temperatures.

The final T-M FSC result is shown in Figure 7 with the results of high-temperature annealing of crystals formed at the low temperature of 70 °C. The temperature jump for subsequent annealing occurred at $1000{\;K\;s}^{-1}$. The melting–recrystallization process up to the annealing temperature $T_{a}$ could have completed with this annealing process. The results shown in Figure 7 were obtained with the longest P, and they show that both C and α are on the respective baselines up to $T_{a}$, followed by pure melting in a small temperature window above $T_{a}$. This behavior confirms the appropriate choice of the baseline for α and that the dip in α is real.

**Figure 8:**
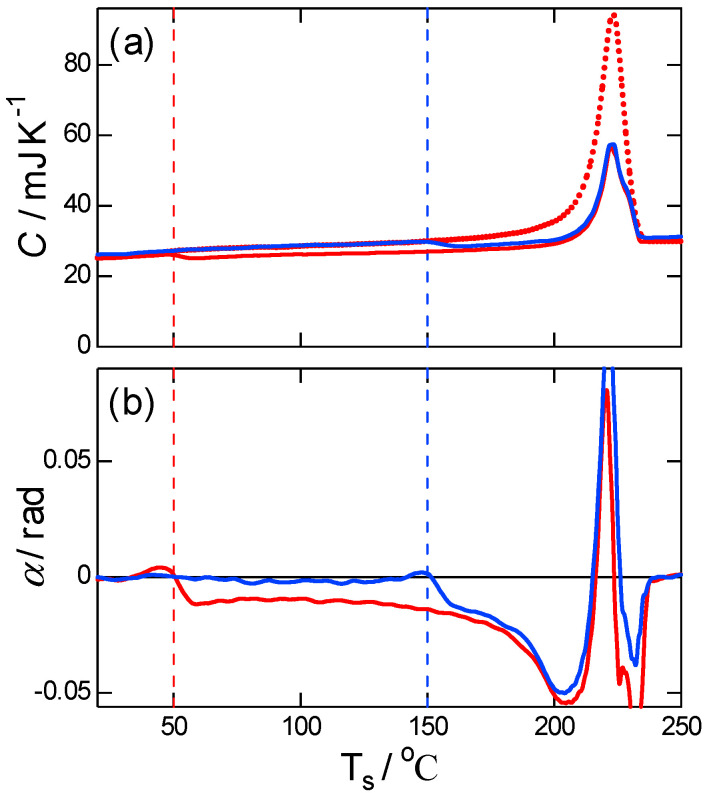
T-M DSC results of (**a**) magnitude and (**b**) phase angle during heating after cold crystallization from the glass at $T_{c}=50\;$°C for 1 min and annealing at $T_{a}=\mathrm{NA},\;150$ °C for 1 min. P=80 s and $\bar{\beta }=3{\;K\;\min}^{-1}$ in the heating-only mode. The dotted line in (**a**) represents −HF/β for the case without annealing. Vertical broken lines represent respective treating temperatures.

Figure 8 shows the corresponding results of T-M DSC obtained at a much slower underlying heating rate $\bar{\beta }$ of $3{\;K\;\min}^{-1}$. The melting is of crystals formed from the glass heated to 50 °C with and without subsequent annealing at 150 °C, which was achieved at a heating scan of $10{\;K\;\min}^{-1}$. The trends are similar to those in Figure 7. This result of PBT is in contrast to that of PET obtained in a previous study [15]. For PET, the response of recrystallization in C and α of T-M DSC was very weak; however, the final melting peak observed in −HF/β shifted to higher temperatures similar that of PBT. Hence, it was concluded that for PET, at heating rates low enough, the shift at low temperatures could be a result of reorganization rather than the melting–recrystallization process. In other words, reorganization exhibits heat flows negligibly smaller than those of melting and recrystallization proceeding as separate processes. In the case of PBT, the recrystallization was pronounced already at low temperatures as evidenced by C and α in Figure 8, and it was more pronounced at higher temperatures, which is similar to the T-M FSC results shown in Figure 7. It is inferred that the faster crystallization of PBT than PET at low temperatures also promoted recrystallization. The rate became higher at higher temperatures in accordance with the negative peak of α in the T-M FSC and DSC; the negative peak indicates higher rates at higher temperatures of exothermic kinetics of recrystallization.

## 4. Conclusions

The melting–recrystallization behavior of PBT was investigated by temperature-modulated scanning calorimetry in both fast-scan and conventional slow-scan rates modes. Recrystallization of PBT simultaneously proceeding with melting was revealed by the response in the effective dynamic heat capacity of complex quantities, such as the peak in the magnitude C, which became larger than −HF/β, and a dip in the phase angle α. The behavior of the PBT was similar to that of PET reported in a previous study, which suggests pronounced recrystallization in the region of double melting peaks over a broad temperature range, which is from just above the crystallization temperature to the main melting peak. However, the behavior of the PBT obtained by the conventional T-M DSC for the temperature modulation at low scan rates was different from that of PET. T-M DSC of the PBT showed pronounced recrystallization over a broad temperature range under a slow heating rate in a way that is similar to that under fast scanning rates, whereas that of PET did not indicate the sign at low temperatures. This difference is attributed to the faster crystallization rate of PBT than PET in orders of magnitudes over the temperature range between the equilibrium melting point and the glass transition temperature. The recrystallization rate is supposed to exhibit the same behavior and to be much more pronounced than in PET. Results of T-M FSC and T-M DSC confirm that the recrystallization of PBT becomes more pronounced at higher temperatures as evidenced by both larger difference between C and −HF/β and larger dip in the phase angle α at higher temperatures. This suggests the occurrence of an activation process mainly driven by the mobility of the polymer chains in a state just after melting and before reaching a random-coiled molten state. There is a need for further experimental and theoretical studies to clarify the behavior of pronounced recrystallization in the melting region during heating.

## Data Availability

Data is contained within the article.
